# Supplementary material for: Apply a Physiologically Based Pharmacokinetic Model to Promote the Development of Enrofloxacin Granules: Predict Withdrawal Interval and Toxicity Dose
Source: Antibiotics (Basel). 2021 Aug 8;10(8):955. doi: 10.3390/antibiotics10080955 (PMC8388861; doi:10.3390/antibiotics10080955)
Supplement: Supplementary file 1 [file antibiotics-10-00955-s001.zip › antibiotics-1321258-supplementary.pdf]

Supplementary Information

Apply a Physiologically Based Pharmacokinetic Model to Promote the Development of Enrofloxacin Granules: Predict Withdrawal Interval and Toxicity Dose

Kaixiang Zhou [1], Aimei Liu [1], Wenjin Ma [1], Lei Sun [1], Kun Mi [1], Xiangyue Xu [1], Samah Attia Algharib [1,2], Shuyu Xie [1,3] and Lingli Huang [1,3,*]

[1] National Reference Laboratory of Veterinary Drug Residues (HZAU) and MAO Key Laboratory for Detection of Veterinary Drug Residues, Wuhan 430070, China; flyingkai@webmail.hzau.edu.cn (K.Z.); 2018302010040@webmail.hzau.edu.cn (A.L.); mawenjin@webmail.hzau.edu.cn (W.M.); sunlei@webmail.hzau.edu.cn (L.S.); mikun@webmail.hzau.edu.cn (K.M.); xuxiangyue@webmail.hzau.edu.cn (X.X.); samah.alghareeb@fvtm.bu.edu.eg (S.A.A.); xieshuyu@mail.hzau.edu.cn (S.X.)

[2] Department of Clinical Pathology, Faculty of Veterinary Medicine, Benha University, Moshtohor, Toukh 13736, Egypt

[3] MOA Laboratory for Risk Assessment of Quality and Safety of Livestock and Poultry Products, Huazhong Agricultural University, Wuhan 430070, China

* Correspondence: huanglingli@mail.hzau.edu.cn; Tel.: +86-027-8728-7186

Content

Table S1. The potential dosage regimen of ENR-SD granules against common intestine bacterial in swine 3

Table S2. The ENR and CIP concentration of tissues in the pig (Mean ± SD, n=3) 3

Table S3. Sensitive parameters identified by the sensitivity analysis.............................3

Table S4. Values and distributions of parameters used in the MC analysis...................4

Table S5. WTs in muscle, fat, liver, and kidney calculated by the PBPK model or the WT 1.4 software method at a dose of 5 mg/kg twice per day for 5 days...4

Table S6. The Viability of pig hepatocytes at different concentration of ENR and CIP (Mean±SD, n=3) 4

Table S7. Physiological parameters used in the PBPK model for ENR in swine 5

Table S8. Chemical-specific parameters used in the PBPK model for ENR in swine....5

Figure S1. The fitting of observed plasma data with predicted data (A) and regression analysis of PBPK model (B). The predicted plasma concentration was well fitted by the observed concentration (R^2=0.9283) 6

Figure S2. The liner linear regression analysis of observed tissue residue concentration of ENR with predicted data (A), observed tissue residue concentration of CIP with predicted data (B), and observed tissue residue concentration of ENR+CIP with predicted data (C). The was predicted tissue residue concentration well fitted by the observed concentration (R^2 > 0.82) 6

Figure S3. The measured WTs of ENR granules in muscle (A), fat (B), liver (C) and kidney (D) of the pig at a dose of 5 mg/kg b.w. twice per day for 5 days. The measured WT of ENR granules in muscle, fat, liver, and kidney of pigs was 3 d, 2 d, 4 d, and 6 d, respectively..7

The method of pig hepatocytes isolation..8

PBPK model code for acslX...9

Table S1. The potential dosage regimen of ENR-SD granules against common intestine bacterial in swine.

Pathogens	MIC_{90} (μg/mL)	Dosage regiment	AUC_{24h} (μg h/mL)	AUCI
Salmonella (n=291)[a,b]	8.0	2.5 mg/kg, twice/day	957	120
Salmonella (n=291)[a,b]	8.0	5.0 mg/kg, once/day	1065	133
E. coli (n=918)[c]	0.25	5.0 mg/kg, once/day	1065	4260
L. intracellularis (n=1)[d]	8.0	5.0 mg/kg, once/day	1065	133
Camp. Jejuni (n=114)[e]	16.0	7.5 mg/kg, once/day	1542	96
Camp. Jejuni (n=114)[e]	16.0	5.0 mg/kg, twice/day	1884	118
Camp. Jejuni (n=114)[e]	16.0	10 mg/kg, once/day	2130	133

Note: [a]Hao et al., 2013; [b]Cao et al., 2017; [c]Wang et al., 2016; [d]Wattanaphansak et al., 2019, [e]Shin et al., 2007; intracellular MIC, only one isolated; AUCI, the ratio of AUC_{24h}/MIC, when AUCI was above 120, the good antibacterial ability against gram-negative bacteria can be expected (Craig, 2001; Schuck et al., 2005); All bacterial were isolated from swine.

Table S2. The ENR and CIP concentration of tissues in the pig (Mean ± SD, n=3).

**Tissues**	**Time (d)**	**ENR (μg/g)**	**CIP (μg/g)**	**Total (μg/g)**	**MRL (μg/g)**
Muscle	0.042	3.13±0.111	0.644±0.108	3.774	0.1
	0.5	1.839±0.076	0.387±0.040	2.226	
	1	0.719±0.007	0.239±0.010	0.958	
	2	0.139±0.027	0.097±0.021	0.236	
	3	0.039±0.006	0.019±0.003	0.058	
	5	< LOD	< LOD	/	
Fat	0.042	1.183±0.108	0.164±0.013	1.347	0.1
	0.5	0.382±0.040	0.111±0.079	0.493	
	1	0.178±0.006	0.061±0.021	0.239	
	2	0.049±0.017	0.036±0.011	0.085	
	3	< LOD	< LOD	/	
	5	< LOD	< LOD	/	
Liver	0.042	6.817±0.047	2.341±0.397	9.158	0.2
	0.5	1.934±0.054	1.350±0.038	3.284	
	1	0.692±0.035	0.843±0.171	1.535	
	2	0.184±0.067	0.602±0.234	0.786	
	3	0.082±0.005	0.124±0.004	0.206	
	5	< LOD	< LOD	/	
Kidney	0.042	6.270±0.504	1.604±0.0.109	7.874	0.3
	0.5	2.852±0.282	1.160±0.097	4.012	
	1	1.301±0.310	1.0±0.194	2.301	
	2	0.432±0.131	0.292±0.119	0.724	
	3	0.221±0.027	0.040±0.012	0.261	
	5	< LOD	< LOD	/	

Note: The MRLs was cited form Codex Alimentarius Commission (CAC), 2017. Maximum residue limits (MRLs) and risk management recommendations (RMRs) for residues of veterinary drugs in foods. CAC/MRL 2-2017. Both the LOD and LOQ were 0.02 μg/mL.

Table S3. Sensitive parameters identified by the sensitivity analysis.

Response	Cmtotalmg	Cftotalmg	Cltotalmg	Cktotalmg
BW	-2.2	-2.0	-1.8	-2.1
Frac	0.37	-0.5	-0.6	-0.4
Kmc	-1.5	-1.4	-1.2	-1.4
Kurinec1	-0.7	-0.8	-1.1	-0.9
Kurinec	-1.5	-1.4	-1.3	-1.6
Pf	0.2	0.8	0.2	0.2
Pf1	/	0.5	/	/
Pk	/	/	/	0.6
Pk1	/	/	/	0.4
Pl	-2.1	-1.9	-1.3	-2.0
Pl1	/	/	0.7	/
Pm	2.0	1.3	1.2	1.3
Pm1	0.6	0.2	0.3	0.2

Note: Only parameters with /NSC/ ≥ 0.2 are presented in the table. Cmtotalmg, Cftotalmg, Cltotalmg, and Cktotalmg represent the final concentration of ENR plus CIP in the muscle, fat, liver and kidney, respectively.

Table S4. Values and distributions of parameters used in the MC analysis.

Parameters	Mean	SD	CV (%)	Lower Bound	Upper Bound
BW	55.0	10.0	0.182	45.0	65.0
Frac	0.35	0.105	0.3	0.245	0.455
Kmc	0.035	0.0105	0.3	0.0245	0.0455
Kurinelc	0.35	0.105	0.3	0.245	0.455
Kurinec	0.2	0.036	0.3	0.184	0.236
Pf	0.6	0.12	0.2	0.48	0.72
Pf1	0.53	0.106	0.2	0.424	0.636
Pk	5.5	1.1	0.2	4.4	6.6
Pk1	5.5	1.1	0.2	4.4	6.6
Pl	3.2	0.64	0.2	2.56	3.64
Pl1	4.3	0.86	0.2	3.44	5.16
Pm	2.5	0.5	0.2	2.0	3.0
Pm1	1.5	0.5	0.2	1.0	2.0

Table S5. WTs in muscle, fat, liver, and kidney calculated by the PBPK model or the WT 1.4 software method at a dose of 5 mg/kg twice per day for 5 days.

Tissues	Predicted WT (d)	Measured WT (d)
Muscle	5	3
Fat	3	2
Liver	6	4
kidney	4	6

Table S6. The Viability of pig hepatocytes at different concentration of ENR and CIP (Mean±SD, n=3).

Concentration (μg/mL)	Viability (%)	
	ENR (%)	CIP (%)
50	106.15±6.83	/
100	96.22±2.92	103.80±1.13
250	45.15±1.95	88.47±7.44
500	13.83±0.56	58.86±3.70
1000	7.27±2.07	7.48±0.20

Table S7. Physiological parameters used in the PBPK model for ENR in swine.

Parameter	Abbreviation	Value[c]
Body weight (kg)	BW	55
Cardiac output (L/h/kg)	QCC	5
**Tissue volume[a]**		
Blood	VBloodC	0.06
Liver	VLC	0.0247
Kidney	VKC	0.004
Muscle	VMC	0.4
Fat	VFC	0.32
Lung	VLuC	0.01
Rest of body	VrestC	0.1813
**Blood flow[b]**		
Liver	QLC	0.2725
Kidney	QKC	0.12
Muscle	QMC	0.251
Fat	QFC	0.1275
Rest of body	QrestC	0.229

Note: [a]fraction of body weight, unitless; [b]fraction of cardiac output, unitless; Lin et al., 2016.

Table S8. Chemical-specific parameters used in the PBPK model for ENR in swine.

Parameter	Abbreviation	Value[a]
Absorption rate constant (/h)		
Gastric emptying	Kst	2.0
Intestinal absorption	Ka	0.55
Intramuscular	Kim	0.0
Subcutaneous	Ksc	0.0
Tissue:plasma partition coefficient for the enrofloxacin (unitless)		
Liver	Pl	3.2
Kidney	Pk	5.5
Muscle	Pm	2.5
Fat	Pf	0.6
Lung	Plu	4.3
Rest of body	Prest	8.0
Tissue:plasma partition coefficient for the main metabolite (unitless)		
Liver	PL1	4.3
Kidney	PK1	5.5
Muscle	PM1	1.5
Fat	PF1	0.53
Lung	Plu1	4.3
Rest of body	Prest1	8.0
*Hepatic metabolic rate [/(h*kg)]*	KmC	0.035
Fraction of enrofloxacin metabolized to the main metabolite (unitless)	Frac	0.35
Percentage of plasma protein binding (unitless)		
Enrofloxacin	PB	0.46
Main metabolite	PB1	0.19
Fecal elimination rate constant (/h)	Kfeces	0.01
Urinary elimination rate constant (L/h/kg)		
Enrofloxacin	KurineC	0.12
Main metabolite	Kurine1C	0.35

Note: [a]Lin et al, 2016.

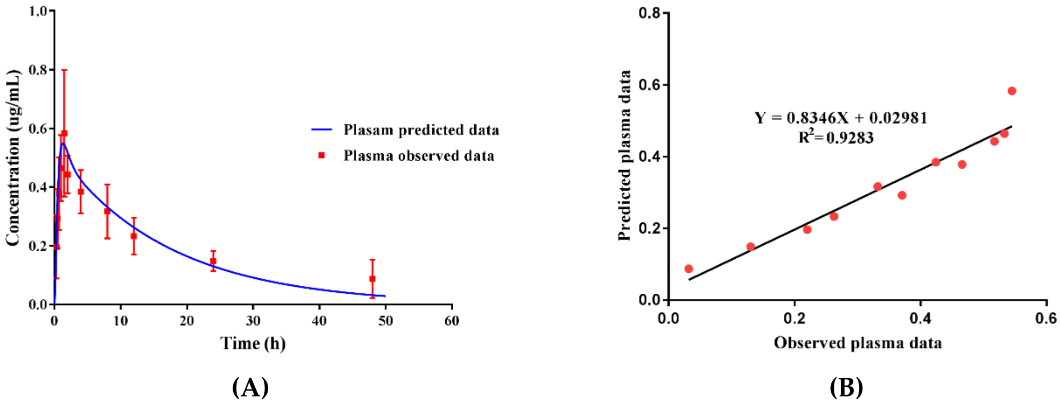

Figure S1. The fitting of observed plasma data with predicted data (A) and regression analysis of PBPK model (B). The predicted plasma concentration was well fitted by the observed concentration (R2=0.9283).

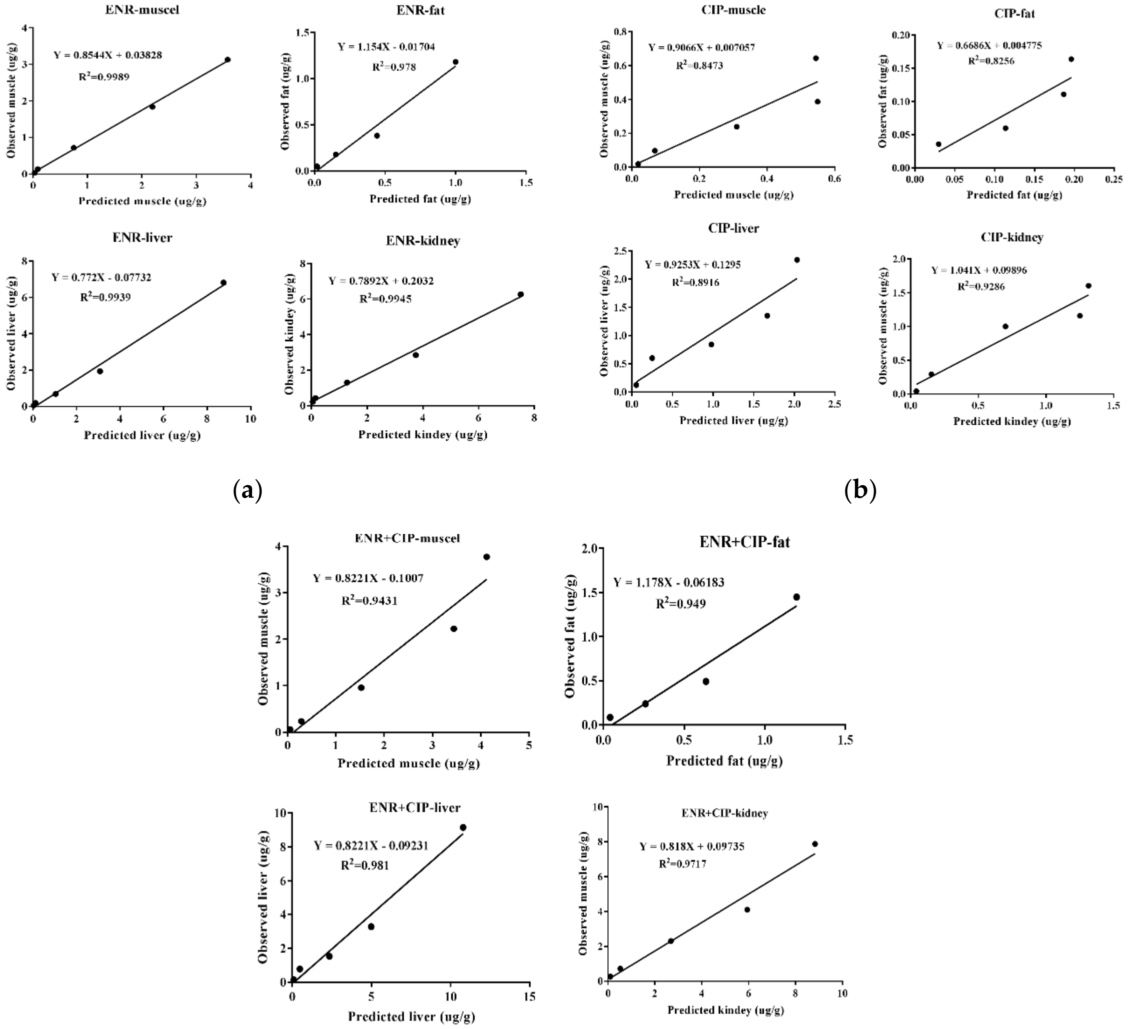

(c)

Figure S2. The liner linear regression analysis of observed tissue residue concentration of ENR with predicted data (A), observed tissue residue concentration of CIP with predicted data (B), and observed tissue residue concentration of ENR+CIP with predicted data (C). The was predicted tissue residue concentration well fitted by the observed concentration (R^2 > 0.82)

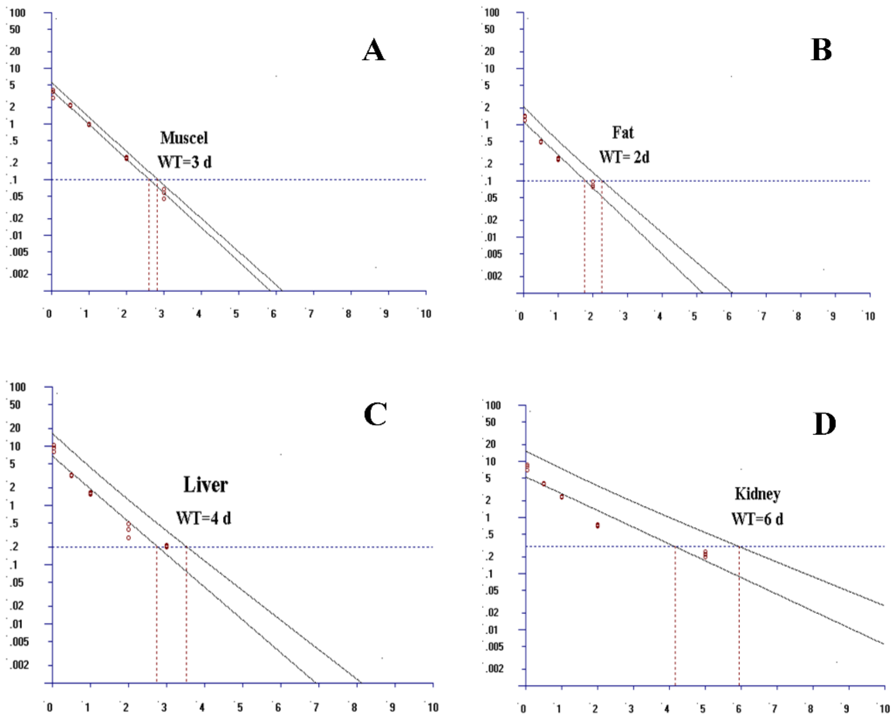

Figure S3. The measured WTs of ENR granules in muscle (A), fat (B), liver (C) and kidney (D) of the pig at a dose of 5 mg/kg b.w. twice per day for 5 days. The measured WT of ENR granules in muscle, fat, liver, and kidney of pigs was 3 d, 2 d, 4 d, and 6 d, respectively.

File S1: The Method of Pig Hepatocytes Isolation

During the residue elimination experiment, the pig was killed by bloodletting. After the abdominal skin was sterilized with 70% alcohol, the abdominal cavity was opened immediately, and a leaf of liver was cut. Then rinse blood repeatedly with ice-cold PBS, and transfer the liver to the super-clean workbench. After that the a 50 mL needle with the perfusion buffer preheated at 37°C was inserted into the larger blood vessels, and slowly inject until the liver is obviously enlarged. After the color becomes light, then inject the perfusion buffer without EDTA at a speed of 30 mL/min, and then perfuse it vertically at about 300 mL. And then, the digestion buffer containing 0.05% collagenase IV preheated at 37°C was replaced and perfused at a speed of 20 mL/min for 20 min. The digested loose liver was quickly placed in a sterilized dish, removing the undigested liver envelope and connective tissue with sterile forceps, add 20 mL of the above 0.08% collagenase IV digestion buffer, cut the liver with forceps, and shake gently. After the cells completely detached, add 20 mL of culture solution containing 10% fetal bovine serum to stop digestion. The above cell precipitates were resuspended and filtered through 200 mesh stainless steel wire mesh to remove residual tissues and cell clumps. Transferred above mixture into a 15 mL centrifuge tube and centrifuged at 800 r/min for 3 min, then resuspended with DMEM culture solution, centrifuged at 800 r/min for 5 min, repeated twice. After discarded the supernatant, the cell precipitates were resuspended with DMEM culture solution for the further culture.

File S2: PBPK Model Code for acslX

Note: The model code below represents a physiologically based pharmacokinetic (PBPK) model for enrofloxacin swine. Parameter values for the studied drugs in swine are provided in Supplementary Tables 2 and Supplementary Tables 3. The PBPK model of other drugs in other animal species can be built based on this code by change the original parameter values.

```
PROGRAM

INITIAL
! code that is executed once at the beginning of a simulation run goes here

!! Physiological parameters
! Blood flow rates (fraction of cardiac output)
CONSTANT QCC = 5 ! Cardiac output index (L/h/kg), also blood flow of lung, from Upton (2008)
CONSTANT QLC = 0.2725 ! liver, average from Buur et al. (2005) and Upton (2008)
CONSTANT QKC = 0.12 ! kidney, average from Buur et al. (2005) and Upton (2008)
CONSTANT QMC = 0.251 ! Muscle, average from Buur et al. (2005) and Upton (2008)
CONSTANT QFC = 0.1275 ! Fat, average from Buur et al. (2005) and Upton (2008)

! Tissue volumes (fraction of body weight)
CONSTANT BW = 55 ! Kg, body weight was study-specific; This value was actual BW in present study
CONSTANT VLC = 0.0247 ! liver, average from Buur et al. (2005) and Upton (2008)
CONSTANT VKC = 0.004 ! Kidneys, average from Buur et al. (2005) and Upton (2008)
CONSTANT VMC = 0.40 ! Muscle, average from Buur et al. (2005) and Upton (2008)
CONSTANT VFC = 0.32 ! Fat, adipose tissue, average from Buur et al. (2005) and Upton (2008)
CONSTANT VLuC = 0.01 ! Lungs, average from Buur et al. (2005) and Upton (2008)
CONSTANT VBloodC = 0.06 ! Blood, average from Buur et al. (2005) and Upton (2008)
CONSTANT VSiC = 0.036 ! Small intestien

!! Mass transfer parameters (Chemical-specific parameters)
! Chemical molecular weights and unit conversion factors, from PubChem
```

```
CONSTANT MW = 359.4 ! g/mol, enrofloxacin

CONSTANT MW1 = 331.34 ! g/mol, ciprofloxacin

CONSTANT MWmol = 2.78 ! umol/mg, enrofloxacin, from mg to umol

CONSTANT MWmg = 0.36 ! mg/umol, enrofloxacin, from umol to mg


! Kinetic constants

! Oral absorption and fecal elimination rate constants (for enrofloxacin)

CONSTANT Kst = 2 ! /h, gastric emptying rate constant

CONSTANT Ka = 0.55 ! /h, intestinal absorption rate constant

CONSTANT Kfeces = 0.01 ! /h, intestinal transit rate constant (fecal elimination rate constant)


! IV infusion/injection rate constants

CONSTANT Timeiv = 0.01 ! h, IV infusion/injection time


! IM absorption rate constants (set parameter value equal to 0.0 when not used in a particular simulation)

CONSTANT Kim = 0.0 ! /h, intramuscular absorption rate constant


! SC absorption rate constants

CONSTANT Ksc = 0.0 ! /h, subcutenous absorption rate constant


! Partition coefficients for enrofloxacins (PC, unitless)

! The values from Buur et al. 2005 were used as initial values for further estimation.

CONSTANT PL = 3.2 ! Liver:plasma PC

CONSTANT PK = 5.5 ! Kidney:plasma PC

CONSTANT PM = 2.5 ! Muscle:plasma PC

CONSTANT PF = 0.6 ! Fat:plasma PC

CONSTANT PLu = 4.3 ! Lung:plasma PC

CONSTANT Prest = 8 ! Rest-of-body:plasma PC


! Partition coefficients for the ciprofloxacin (usually designated as the ciprofloxacin) (PC, unitless)

CONSTANT PL1 = 4.3 ! Liver:plasma PC

CONSTANT PK1 = 5.5 ! Kidney:plasma PC
```

```
CONSTANT PM1 = 1.5 ! Muscle:plasma PC

CONSTANT PF1 = 0.53 ! Fat:plasma PC

CONSTANT PLu1 = 4.3 ! Lung:plasma PC

CONSTANT Prest1 = 8 ! Rest-of-body:plasma PC


! Percentage plasma protein binding (unitless), Buur et al. (2005)

CONSTANT PB = 0.46 ! Percentage of enrofloxacin bound to plasma proteins

CONSTANT PB1 = 0.19 ! Percentage of the ciprofloxacin bound to plasma proteins


! Metabolic rate constants

CONSTANT KmC = 0.035 ! /(h*kg), liver metabolic rate constant of the enrofloxacin

CONSTANT Frac = 0.35 ! Unitless, fraction of enrofloxacin metabolized to the ciprofloxacin


! Urinary elimination rate constants

CONSTANT KurineC = 0.12 ! L/h/kg, for enrofloxacin

CONSTANT Kurine1C = 0.35 ! L/h/kg, for the ciprofloxacin

CONSTANT PDOSEoral = 130 ! mg/kg

CONSTANT PDOSEiv = 0 ! mg/kg

CONSTANT PDOSEim = 0 ! mg/kg

CONSTANT PDOSEsc = 0 ! mg/kg


END ! INITIAL

DYNAMIC


ALGORITHM IALG = 2

NSTEPS NSTP = 10

MAXTERVAL MAXT = 1.0e9

MINTERVAL MINT = 1.0e-9

CINTERVAL CINT = 0.1


DERIVATIVE

! code for calulating the derivative goes here
```

```
! Cardiac output and blood flows to tissues (L/h)
QC=QCC*BW ! Cardiac output
QL=QLC*QC ! Blood flow to the liver
QK=QKC*QC ! Blood flow to the kidney
QM=QMC*QC ! Blood flow to the muscle
QF=QFC*QC ! Blood flow to the fat
Qrest = QC-QL-QK-QM-QF ! Blood flow to the rest of body

! Tissue volumes (L)
VL=VLC*BW ! Liver
VK=VKC*BW ! Kidney
VM=VMC*BW ! Muscle
VF=VFC*BW ! Fat
VLu=VLuC*BW ! Lung
VBlood=VBloodC*BW ! Blood
Vven=VBlood*0.74 ! Venous blood
Vart=VBlood*0.26 ! Arterial blood
Vrest = BW-VL-VK-VM-VF-VLu-VBlood ! Rest of body
VSi=VSiC*BW ! Small intestine

! Dosing amounts (mg converted to umol)
DOSEoral=PDOSEoral*BW*MWmol ! umol
DOSEiv=PDOSEiv*BW*MWmol ! umol
DOSEim=PDOSEim*BW*MWmol ! umol
DOSEsc=PDOSEsc*BW*MWmol ! umol

! Multiple oral dosing using the PULSE/EXPOSURE function
CONSTANT tlen = 0.001 ! Length of exposure, oral, iv, im, or sc (h/day)
CONSTANT tinterval = 12 ! administration interval, varied dependent on the exposure paradigm (h)
CONSTANT Dstart = 0.0 ! Initiation day of exposure (day)
CONSTANT Dstop = 5 ! Termination day of exposure (day)
CONSTANT MAXT = 1.0 ! maximum comm. interval
```

```
CONSTANT CINTC = 0.1 ! Communication interval

CINT = CINTC ! Communication interval

Tsim=TSTOP*24 ! Tstop in hours

DS=Dstart*24 ! Initiation time point of exposure (h)

Doff=(Dstop-Dstart)*24 ! Exposure duration (h)

TimeOn=Dstart*24 ! Initiation time point of exposure (h)

TimeOff=Dstop*24+tlen ! Terminatison time point of exposure (h)

Exposure=PULSE(0,tinterval,tlen)*PULSE(DS,Tsim,Doff) ! Exposure paradigm

RDOSEoral=(DOSEoral/tlen)*Exposure ! Administrataion rate

RAST=RDOSEoral-Kst*AST ! Rate in the stomach

AST=Integ(RAST,0)!0.0 or Doseoral if the initial dose is twice as the subsequent dose.

RAI=Kst*AST-Ka*AI-Kfeces*AI ! Rate in the intestine

Rfeces=Kfeces*AI ! Fecal elimination rate

Afeces=Integ(Rfeces,0.0) ! Amount eliminated through feces

AI=Integ(RAI,0.0) ! Amount in the intestine

CAI=AI/VSi ! Concentration of the enrofloxacin in small intestine, umol/L

CAImg=AI*MWmg ! Concentration of the total enrofloxacin in small intestine, unit conversion from umol/L to mg/L (ug/g)

RAO=Ka*AI ! Oral absorption rate

AAO=Integ(RAO,0.0) ! Amount absorbed


! Single IV dosing to the venous

IVR=DOSEiv/timeiv

RIV=IVR*(1.0-step(timeiv)) ! Intravenous injection rate

AIV=Integ(RIV,0.0) ! Amount injected


! Single IM exposure

Rim=Kim*Aimsite ! Intramuscular absorption rate

Aim=Integ(Rim,0.0) ! Amount absorbed via IM route

Rimsite=-Kim*Aimsite ! Rate of changes in the amount of the drug in the injection site

Aimsite=Integ(Rimsite,Doseim) ! Amount of the drug remained in the injection site


! Multiple IM exposure (if needed)
```

```
!RDOSEim=(DOSEim/tlen)*Exposure
!Rimsite=RDOSEim-Kim*Aimsite
!Aimsite=Integ(Rimsite,0.0)
!Rim=Kim*Aimsite
!Aim=Integ(Rim,0.0)

! Single SC exposure
Rsc=Ksc*Ascsite ! Subcutaneous absorption rate
Asc=Integ(Rsc,0.0) ! Amount absorbed via SC route
Rscsite=-Ksc*Ascsite ! Rate of changes in the amount of the drug in the injection site
Ascsite=Integ(Rscsite,Dosesc) ! Amount of the drug remained in the injection site

! Metabolic rate
Km=KmC*BW ! h-1
! Urinary elimination rates
Kurine=KurineC*BW ! L/h, for the enrofloxacin
Kurine1=Kurine1C*BW ! L/h, for the ciprofloxacin

! ********Sub-model for the enrofloxacin (enrofloxacin)***************************
! Venous blood/plasma
RV=QL*CVL+QK*CVK+QM*CVM+QF*CVF+Qrest*CVrest+Riv+Rim+Rsc-QC*CV ! Rate, umol/h
AV=Integ(RV,0.0) ! Amount, umol
CV=AV/Vven ! Concentration of the total enrofloxacin (free plus bound), umol/L
CVfree=CV*(1-PB) ! Concentration of the enrofloxacin that is free, umol/L
CVbound=CV*PB ! Concentration of the enrofloxacin that is bound, umol/L
CVmg=CV*MWmg ! Concentration of the total enrofloxacin (free plus bound), unit conversion from umol/L to mg/L (ug/g)

! Arterial blood/plasma
RA=QC*CVLu-QC*CAfree ! Rate, umol/h
AA=Integ(RA,0.0) ! Amount, umol
CA=AA/Vart ! Concentration of the total enrofloxacin (free plus bound), umol/L
CAfree=CA*(1-PB) ! Concentration of the enrofloxacin that is free, umol/L
```

CAbound=CA*PB ! Concentration of the enrofloxacin that is bound, umol/L

ABlood=AV+AA ! Amount of the total drug in the blood, umol

! Lung compartment

RALu=QC*(CV-CVLu) ! Rate, umol/h

ALu=Integ(RALu,0.0) ! Amount, umol

CLu=ALu/VLu ! Concentration of the total enrofloxacin in the lung, umol/L

CVLu=CLu/PLu ! Concentration of the total enrofloxacin in venous blood drained from the lung, umol/L

! Liver compartment

RL=QL*(CAfree-CVL)+RAO-Rmet ! Rate, umol/h

AL=Integ(RL,0.0) ! Amount, umol

CL=AL/VL ! Concentration of the total enrofloxacin in the liver, umol/L

CVL=CL/PL ! Concentration of the total enrofloxacin in the venous blood drained from the liver, umol/L

CLmg=CL*MWmg ! Concentration of the total enrofloxacin in the liver, mg/L (ug/g)

! Metabolism of the enrofloxacin in the liver compartment

Rmet=Km*CL*VL ! Total hepatic metabolic rate, umol/h

Rmet1=Rmet*Frac ! Hepatic metabolic rate to the ciprofloxacin, umol/h

Rmet2=Rmet*(1-Frac) ! Hepatic metabolic rate to other minor metabolites, umol/h

Amet=Integ(Rmet,0.0) ! Amount of the enrofloxacin that is metabolized in the liver, umol

Amet1=Integ(Rmet1,0.0) ! Amount of the ciprofloxacin that is produced in the liver, umol

Amet2=Integ(Rmet2,0.0) ! Amount of other minor metabolites that are produced in the liver, umol

! Kidney compartment

RK=QK*(CAfree-CVK)-Rurine ! Rate, umol/h

AK=Integ(RK,0.0) ! Amount, umol

CK=AK/VK ! Concentration of the total enrofloxacin in the kidney, umol/L

CVK=CK/PK ! Concentration of the total enrofloxacin in the venous blood drained from the kidney, umol/L

Ckmg=Ck*MWmg ! Concentration of the total enrofloxacin in the kidney, mg/L (ug/g)

! Urinary excretion of the enrofloxacin

```
Rurine=Kurine*CVK ! Rate, umol/h
Aurine=Integ(Rurine,0.0) ! Amount, umol

! Muscle compartment
RM=QM*(CAfree-CVM) ! Rate, umol/h
AM=Integ(RM,0.0) ! Amount, umol
CM=AM/VM ! Concentration of the total enrofloxacin in the muscle, umol/L
CVM=CM/PM ! Concentration of the total enrofloxacin in the venous blood drained from the muscle, umol/L
CMmg=CM*MWmg ! Concentration of the total enrofloxacin in the muscle, mg/L (ug/g)

! Fat compartment
RF=QF*(CAfree-CVF) ! Rate, umol/h
AF=Integ(RF,0.0) ! Amount, umol
CF=AF/VF ! Concentration of the total enrofloxacin in the fat, umol/L
CVF=CF/PF ! Concentration of the total enrofloxacin in the venous blood drained from the fat, umol/L
CFmg=CF*MWmg ! Concentration of the total enrofloxacin in the fat, mg/L (ug/g)

! Rest-of-body compartment
Rrest=Qrest*(CAfree-CVrest) ! Rate, umol/h
Arest=Integ(Rrest,0.0) ! Amount, umol
Crest=Arest/Vrest ! Concentration of the total enrofloxacin in the rest-of-body, umol/L
CVrest=Crest/Prest ! Concentration of the total enrofloxacin in the venous blood drained from the rest-of-body, umol/L

! Mass balance for the enrofloxacin
Qbal=QC-QL-QK-QM-QF-Qrest ! Blood flow balance
Tmass=ABlood+AL+AK+AM+AF+Arest+ALu+Aurine+Amet ! Total amount in the body, umol
Bal=AAO+AIV+AIM+ASC-Tmass ! Mass balance, input minus output should be equal to zero at all time

! **********Sub-model for the ciprofloxacin (usually the ciprofloxacin)*************
! Venous blood/plasma
RV1=QL*CVL1+QK*CVK1+QM*CVM1+QF*CVF1+Qrest*CVrest1-QC*CV1 ! Rate, umol/h
AV1=Integ(RV1,0.0) ! Amount, umol
```

```
CV1=AV1/Vven ! Concentration of the ciprofloxacin (free plus bound), umol/L

CV1free=CV1*(1-PB1) ! Concentration of the ciprofloxacin that is free, umol/L

CV1bound=CV1*PB1 ! Concentration of the ciprofloxacin that is bound, umol/L

CV1mg=CV1*MW1mg ! Concentration of the ciprofloxacin (free plus bound), unit conversion from umol/L to mg/L (ug/g)

CVtotalmg=CVmg+CV1mg ! Concentration of the enrofloxacin plus the ciprofloxacin, mg/L (ug/g)


! Arterial blood/plasma

RA1=QC*CVLu1-QC*CA1free ! Rate, umol/h

AA1=Integ(RA1,0.0) ! Amount, umol/h

CA1=AA1/Vart ! Concentration of the ciprofloxacin (free plus bound), umol/L

CA1free=CA1*(1-PB1) ! Concentration of the ciprofloxacin that is free, umol/L

CA1bound=CA1*PB1 ! Concentration of the ciprofloxacin that is bound, umol/L

ABlood1=AV1+AA1 ! Amount of the ciprofloxacin in the blood, umol


! Lung compartment

RALu1=QC*(CV1-CVLu1) ! Rate, umol/h

ALu1=Integ(RALu1,0.0) ! Amount, umol

CLu1=ALu1/VLu ! Concentration of the ciprofloxacin in the lung, umol/L

CVLu1=CLu1/PLu ! Concentration of the ciprofloxacin in venous blood drained from the lung, umol/L


! Liver compartment

RL1=QL*(CA1free-CVL1)+Rmet1 ! Rate, umol/h

AL1=Integ(RL1,0.0) ! Amount, umol

CL1=AL1/VL ! Concentration of the ciprofloxacin in the liver, umol/L

CVL1=CL1/PL1 ! Concentration of the ciprofloxacin in venous blood drained from the liver, umol/L

CL1mg=CL1*MW1mg ! Concentration of the ciprofloxacin in the liver, mg/L (ug/g)

CLtotalmg=CL1mg+CLmg ! Concentration of the enrofloxacin plus the ciprofloxacin, mg/L (ug/g)


! Kidney compartment

RK1=QK*(CA1free-CVK1)-Rurine1 ! Rate, umol/h

AK1=Integ(RK1,0.0) ! Amount, umol

CK1=AK1/VK ! Concentration of the ciprofloxacin in the kidney, umol/L
```

CVK1=CK1/PK1 ! Concentration of the ciprofloxacin in venous blood drained from the kidney, umol/L

CK1mg=CK1*MW1mg ! Concentration of the ciprofloxacin in the kidney, mg/L (ug/g)

CKtotalmg=CK1mg+CKmg ! Concentration of the enrofloxacin plus the ciprofloxacin, mg/L (ug/g)

! Urinary excretion of the ciprofloxacin

Rurine1=Kurine1*CVK1 ! Rate, umol/h

Aurine1=Integ(Rurine1,0.0) ! Amount, umol

! Muscle compartment

RM1=QM*(CA1free-CVM1) ! Rate, umol/h

AM1=Integ(RM1,0.0) ! Amount, umol

CM1=AM1/VM ! Concentration of the ciprofloxacin in the muscle, umol/L

CVM1=CM1/PM1 ! Concentration of the ciprofloxacin in venous blood drained from the muscle, umol/L

CM1mg=CM1*MW1mg ! Concentration of the ciprofloxacin in the muscle, mg/L (ug/g)

CMtotalmg=CM1mg+CMmg ! Concentration of the enrofloxacin plus the ciprofloxacin, mg/L (ug/g)

! Fat compartment

RF1=QF*(CA1free-CVF1) ! Rate, umol/h

AF1=Integ(RF1,0.0) ! Amount, umol

CF1=AF1/VF ! Concentration of the ciprofloxacin in the fat, umol/L

CVF1=CF1/PF1 ! Concentration of the ciprofloxacin in venous blood drained from the fat, umol/L

CF1mg=CF1*MW1mg ! Concentration of the ciprofloxacin in the fat, mg/L (ug/g)

CFtotalmg=CF1mg+CFmg ! Concentration of the enrofloxacin plus the ciprofloxacin, mg/L (ug/g)

! Rest-of-body compartment

Rrest1=Qrest*(CA1free-CVrest1) ! Rate, umol/h

Arest1=Integ(Rrest1,0.0) ! Amount, umol

Crest1=Arest1/Vrest ! Concentration of the ciprofloxacin in the rest-of-body, umol/L

CVrest1=Crest1/Prest1 ! Concentration of the ciprofloxacin in venous blood drained from the rest-of-body, umol/L

! Mass balance for the ciprofloxacin

Tmass1=ABlood1+AL1+AK1+AM1+AF1+Arest1+ALu1+Aurine1

```
Bal1=Amet1-Tmass1 ! Input minus output should be equal to zero at all time

END ! DERIVATIVE
! Add discrete events here as needed
! DISCRETE
! END

! code that is executed once at each communication interval goes here
CONSTANT TSTOP = 228
TERMT (T .GE. TSTOP, 'checked on communication interval: REACHED TSTOP')

END ! DYNAMIC
TERMINAL
! code that is executed once at the end of a simulation run goes here
END ! TERMINAL
END ! PROGRAM
```
